# Intensifying vaccine production

**DOI:** 10.2471/BLT.20.020520

**Published:** 2020-05-01

## Abstract

The COVID-19 pandemic has started a race to develop new vaccines. Making sure enough is made to meet demand will require manufacturing innovation. Tatum Anderson reports.

The COVID-19 pandemic has focused attention on the need for a SARS-CoV-2 vaccine, at least 43 of which are currently in development. After the necessary trials in humans, the hope is that one or more of these candidate vaccines will emerge as a safe and cost-effective response to the pandemic – possibly in the next twelve months.

But at that point stakeholders will be faced with another challenge: how to manufacture and distribute enough quality vaccine to meet global demand.

It is broadly accepted that arranging to ensure high volume production should start now. Some governments are already negotiating deals with vaccine manufacturers.

In March, for example, the government of the United States of America (USA), signed a US$1 billion deal with the pharmaceutical company Johnson & Johnson to develop and manufacture more than 1 billion doses of a candidate vaccine.

The fact that one of the first SARS-CoV-2 vaccine deals to be struck is between the richest country in the world and one of the world’s biggest pharmaceutical companies is indicative of the financial stakes typically involved in vaccine development and manufacture.

According to a study published in the July 2017 issue of *Vaccine*, in the USA, it costs between US$ 50 million to US$ 500 million to set up a facility to produce monovalent vaccines and as much as US$ 700 million for polyvalent vaccines. Because running such facilities is complex, there is also a need for a skilled workforce to ensure operations.

As a result, vaccine production is generally undertaken by well-resourced manufacturers serving clients in countries where access to skilled workers is not a problem. Smaller manufacturers and manufacturers located in resource poor settings are shut out.

“A significant issue at any time, the inequities inherent in vaccine production become a serious problem when ensuring global access to an effective vaccine is vital, as is the case in the developing COVID-19 pandemic,” says Erin Sparrow, an infectious disease expert that the World Health Organization (WHO).

Establishing vaccine manufacturing is not only expensive, it is typically slow, taking seven years to design, build, validate, and commence commercial manufacturing in a 3-product polyvalent vaccine facility according to the above-mentioned study.

One possible approach to addressing these challenges is to apply what has come to be known as process intensification, a manufacturing technique designed to reduce the time and space required to make vaccines, while also reducing processing complexity.

“Process intensification has the potential to make vaccines and biologicals production cheaper by orders of magnitude,” says Martin Friede, coordinator of the Initiative for Vaccine Research at the World Health Organization (WHO). “It can also reduce operator-dependent risks, which makes it suitable for countries lacking the workforce skills needed to run traditional plants,” he adds.

“Process intensification has the potential to make vaccine and biologicals production cheaper by orders of magnitude.”

Martin Friede

Process intensification boils down to two key concepts: densification and chaining.

“Densification is achieved by redesigning or indeed inventing equipment that takes up less space,” explains José Castillo, chief technical officer at Univercells, a Belgian biotechnology company, which has developed a 50-liter bioreactor using a three-dimensional matrix of polyethylene fibers that can grow as much as a traditional 1000-liter bioreactor.

Densification is also achieved outside the bioreactor, which, because it is so small, can be contained inside a biosafety cabinet or a slightly larger sterile container called an isolator. This means that processes usually run in contamination-free ‘clean rooms’ which are expensive to build and maintain, can be executed in an ordinary room.

Univercells has also developed a platform that combines chaining (continuous or semi-continuous processing) and automation, enabling manufacture with an extremely reduced footprint. “A facility with a footprint of 6m² can replace traditional equipment that would take up 120m²,” Castillo says.

Netherlands-based biotech company Batavia Biosciences, is also working on process intensification, using technology developed by Univercells to develop a platform that allows for streamlined, semi-continuous processing.

“With semi-continuous processing, the output from one step flows almost continuously into the next without waiting,” says Ahd Hamidi, head of Global Health projects at the company. “A process that usually takes a week, can take just a few days, lowering operational costs.”

The platforms developed by Batavia and Univercells are both ‘plug and play’, relying on prefabricated units. Manufacturers can also change elements according to the product they are making. Because the processes are essentially closed, there is also less likelihood of contamination. “Chaining reduces the safety issues and batch-to-batch variations associated with physical handling,” Hamidi explains.

Since 2016, Univercells and Batavia have been working together on a project funded through a Bill & Melinda Gates Foundation Grand Challenges Grant. The foundation asked groups to design innovative platform vaccine manufacturing technologies to reduce vaccine costs to less than US$ 0.15 per dose and at the relatively modest volume of 40 million doses per year.

The idea behind the challenge was to encourage global vaccine supply from multiple small facilities while also putting the stress on innovation. “We wanted to ensure that any cost savings would derive from innovative technology solutions, rather than economies of scale,” said Stephen Hadley, senior programme officer for Vaccine Development and Surveillance at the foundation.

Univercells and Batavia spent two years designing a manufacturing process for Sabin inactivated polio vaccine (sIPV) and achieved an estimated cost per dose for 40 million doses for less than US$ 0.30. While twice the target set by the foundation, it is a fifth of the current UNICEF price for this vaccine.

“We estimate that the vaccine could be produced in a micro-facility, costing approximately US$ 30 million and capable of delivering between 40-50 million trivalent doses per year,” Hamidi says, adding that the investment required to go into production at that scale would typically be between US$ 100 – 150 million.

“Chaining reduces the safety issues and batch-to-batch variations associated with physical handling.”

Ahd Hamidi

Batavia has already begun discussions with manufacturers, and Hamidi is hopeful that some will be using the platform technology as part of feasibility studies in 2020.

According to Castillo, Ecuador has already committed to using the technology, which it will install in seventeen sterile shipping containers at the University Hospital of Cuenca, in Ecuador’s third-largest city. The facility will make monoclonal antibodies to treat rheumatoid arthritis.

As exciting as the Ecuador project may be, for the time being it is the prospect of applying process intensification as part of pandemic response that is likely to draw most attention.

The Coalition for Epidemic Preparedness Innovations (CEPI) is already working with Batavia. A global alliance financing and coordinating the development of vaccines against emerging infectious diseases, CEPI is leading the financing for research and development of COVID-19 vaccines. With CEPI, Batavia is designing processes using the Univercells technology to manufacture vaccine candidates against Nipah and Lassa fevers.

In March 2020, the International AIDS Vaccine Initiative (IAVI), a nonprofit scientific research organization, announced a partnership with Batavia to develop vaccines for emerging infectious diseases, including viral hemorrhagic fevers. The partners will also be working on the development of a vaccine candidate for COVID-19.

Batavia and Univercells have also received a second grant from the Bill & Melinda Gates Foundation to work on the development of platforms to increase the availability of measles and rubella vaccines in low- and middle-income countries.

Of course, Univercells and Batavia are not the only companies working on process intensification. Indeed, most of the leading players involved in vaccine and biologics process or product development are implementing or seeking to implement aspects of process intensification.

Examples include pharmaceutical companies Janssen and Merck – Janssen using high-yield cell substrates to boost output, Merck employing single-use membrane chromatography to speed production and save space.

Other players are working on ‘plug and play’ production platforms, such as Cytiva, a technologies and services provider, which has developed a prefabricated modular manufacturing facility that can produce a variety of vaccines and therapeutics, including monoclonal antibodies.

“[Our solutions] are designed to be ready-to-run in 14-18 months, allowing manufacturers to add production capacity to meet demand quickly,” says Daria Donati, Cytiva’s director of business development and innovation.

While 14-18 months may not sound very fast, it is a great deal quicker than the multi-year timelines required for most manufacturing facilities. For WHO’s Sparrow, it is this compressed timeline that is of interest in the context of pandemic response. “You could in theory have a small facility that could be switched on relatively quickly in the event of an outbreak and used to produce a vaccine,” she says.

Process intensification will not solve all the problems faced in getting biologics and vaccines from the laboratory to the people who need them, having no impact on the time taken to run clinical trials or get regulatory approval, for example. However, it looks likely to accelerate the progress of vaccines along the value chain, at what has been an important pinch point, while allowing countries to establish links in that chain where none previously existed.

**Figure:**
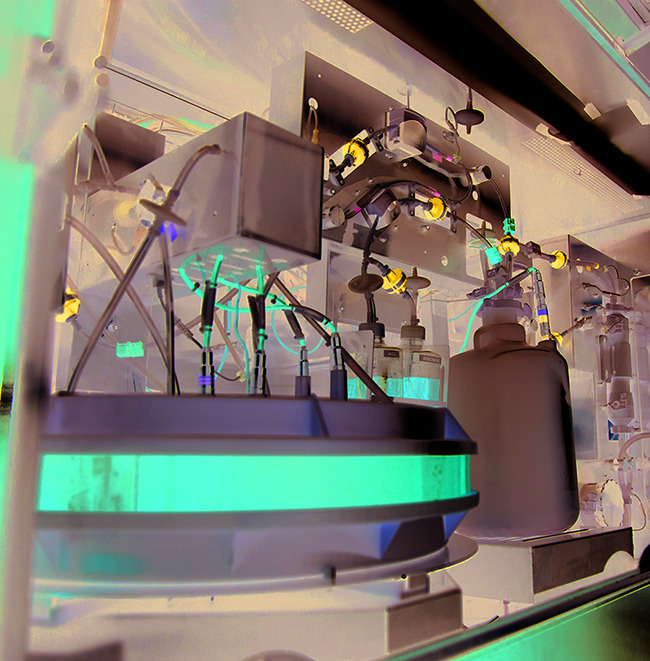
A small-footprint, high-output bioreactor

Univercells /Didier Ropers

**Figure:**
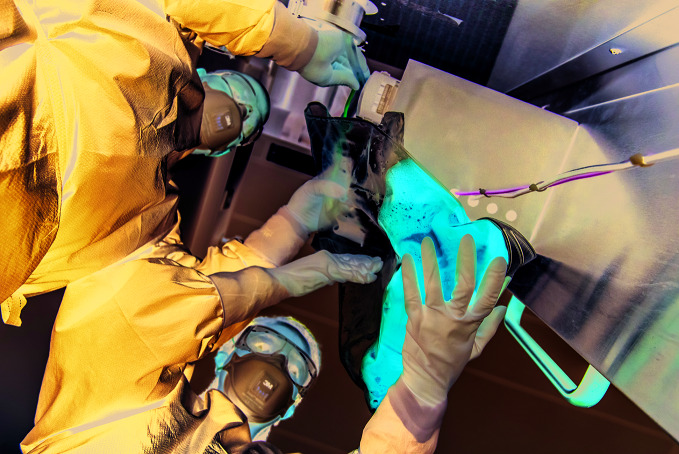
Technicians connect medium supply to a micro-facility inside an isolator

Batavia Biosciences/Gerke van de Hoef

